# Cortical complexity in world trade center responders with chronic posttraumatic stress disorder

**DOI:** 10.1038/s41398-021-01719-7

**Published:** 2021-11-23

**Authors:** Minos Kritikos, Sean A. P. Clouston, Chuan Huang, Alison C. Pellecchia, Stephanie Mejia-Santiago, Melissa A. Carr, Roman Kotov, Roberto G. Lucchini, Samuel E. Gandy, Evelyn J. Bromet, Benjamin J. Luft

**Affiliations:** 1 Program in Public Health and Department of Family, Population, and Preventive Medicine, Renaissance School of Medicine at Stony Brook University, Stony Brook, NY USA; 2 Department of Radiology, Renaissance School of Medicine at Stony Brook, Stony Brook, NY USA; 3 Department of Psychiatry, Renaissance School of Medicine at Stony Brook University, Stony Brook, NY USA; 4 World Trade Center Health and Wellness Program, Renaissance School of Medicine at Stony Brook University, Stony Brook, NY USA; 5 Department of Environmental Health Sciences, Robert Stempel School of Public Health, Florida International University, Miami, FL USA; 6 Department of Neurology, Icahn School of Medicine at Mount Sinai, New York, NY USA; 7 Department of Psychiatry and Mount Sinai Alzheimer’s Disease Research Center, Icahn School of Medicine at Mount Sinai, New York, NY USA; 8 Department of Medicine, Renaissance School of Medicine at Stony Brook University, Stony Brook, NY USA

**Keywords:** Human behaviour, Neuroscience, Diagnostic markers, Psychiatric disorders

## Abstract

Approximately 23% of World Trade Center (WTC) responders are experiencing chronic posttraumatic stress disorder (PTSD) associated with their exposures at the WTC following the terrorist attacks of 9/11/2001, which has been demonstrated to be a risk factor for cognitive impairment raising concerns regarding their brain health. Cortical complexity, as measured by analyzing Fractal Dimension (FD) from T_1_ MRI brain images, has been reported to be reduced in a variety of psychiatric and neurological conditions. In this report, we hypothesized that FD would be also reduced in a case-control sample of 99 WTC responders as a result of WTC-related PTSD. The results of our surface-based morphometry cluster analysis found alterations in vertex clusters of complexity in WTC responders with PTSD, with marked reductions in regions within the frontal, parietal, and temporal cortices, in addition to whole-brain absolute bilateral and unilateral complexity. Furthermore, region of interest analysis identified that the magnitude of changes in regional FD severity was associated with increased PTSD symptoms (reexperiencing, avoidance, hyperarousal, negative affect) severity. This study confirms prior findings on FD and psychiatric disorders and extends our understanding of FD associations with posttraumatic symptom severity. The complex and traumatic experiences that led to WTC-related PTSD were associated with reductions in cortical complexity. Future work is needed to determine whether reduced cortical complexity arose prior to, or concurrently with, onset of PTSD.

## Introduction

Responders exposed to the terrorist attacks on the World Trade Center (WTC) on September 11^th^, 2001, experienced severe psychological trauma [1–22]. In the years after 9/11, approximately 23% of these responders continued reported symptoms of chronic post-traumatic stress disorder [PTSD] [1, 22, 23]. Several studies by our group [2, 3, 24, 25] and other investigators [26–29] have shown that PTSD is a risk factor for cognitive impairment (CI). Neuroimaging studies have identified neurobiological changes in the brains of patients with PTSD [30]. Moreover, studies have identified negative association between PTSD severity and cortical thickness (CTX) with combat exposed veterans with PTSD [31], in veterans exposed to early life trauma [32], prolonged single trauma exposure survivors with PTSD [33], increased combat exposure severity [34], and in veterans with increased lifetime burden of PTSD [35]. However, our prior study of WTC responders with long-term PTSD examined cortical thickness and did not find patterns that differentiated PTSD from non-PTSD patients. This was surprising, however difficulty replicating findings across studies can be due to differences in exposure severity and recency, sample characteristics, methods of assessing PTSD, and focus of the imaging studies themselves. Therefore, we sought to identify alternative neurobiological changes that might occur in WTC-responders with PTSD.

Two recent studies identified reductions in gyrification associated with PTSD severity [36, 37], suggesting that PTSD may induce morphological changes. A more comprehensive measure of cortical complexity than gyrification is fractal dimension (FD). T_1_-weighted MRI image cortical complexity can be measured through the extraction and quantification of a FD metric, which can elucidate the size and complexity of cerebral surfaces. Volumetric structural MRI images permit the 3D reconstruction of cortical surfaces in order to perform 3D-FD “box-counting” of these surfaces down to a spatial scale of 3 mm, which corresponds well with measures of CTX [38], or down to 1 mm when using a different implementation [39]. FD analysis techniques can go beyond just estimating CTX, as FD additionally measures gyrification and sulcal depth of the cortical ribbon as a single quantifiable index value [40, 41]. FD analysis is a promising method to study inherent irregularities of cerebral geometry in comparison to measures relying on integral Euclidian geometry, as nonintegral FD follows the more practical geometry found in nature and can be applied to the complicated, irregular and fractal convolutedness of cerebral surfaces [42, 43].

FD has yet to be studied in populations with PTSD, but recent studies in psychiatry have begun to examine cortical complexity. For example, individuals with chronic psychiatric conditions, including obsessive-compulsive disorder, anorexia nervosa and schizophrenia [44–48], have all identified unique patterns of reductions in cortical complexity. Additionally, reductions in cortical complexity have also been identified in advanced neurological disorders that exhibit concomitant psychiatric symptoms, such as frontal lobe epilepsy [49], Alzheimer’s disease [50, 51], Spinocerebellar Ataxia Type 2 [52], small vessel disease with CI [53], multiple sclerosis [54, 55], Amyotrophic Lateral Sclerosis [56], frontotemporal dementia [FTD] [57], and also the aging brain [39, 58–61]. Therefore, FD presents as a novel and useful measurement of cortical complexity in psychiatrically affected populations and in this paper, we examined WTC responders with and without long-term PTSD to determine if those with PTSD would exhibit reductions in cortical complexity.

PTSD is a heterogenous condition often comorbid with depression, anxiety, and cognitive impairment that involves complex memory, emotional, and behavioral processes [62], and encompasses distinct symptom domains including reexperiencing traumatic events, effortful avoidance, hyperarousal, and emotional numbing/negative affect [63]. Prior work has noted that reexperiencing symptoms are more consistently associated with risk of aging-related conditions [7, 24, 25, 64–66], while serologic analyses have found that avoidance was identified as a key symptom to older DNA methylation age estimates [67] and transcriptomics dysregulation [68].

The primary aim of this paper was to study cortical complexity, measured as FD, in the whole brain, each hemisphere, and by region of interest (ROI) parcellation, in a sample of 99 WTC responders with and without long-term PTSD. A secondary aim of this study was to generate outcome estimates of cortical complexity alterations by total PTSD symptom severity, and by the severity each of the four individual symptom domains, in affected regions of interest (ROIs) while controlling for cortical thickness in respective ROIs and for differences in comorbidities. These adjustments were applied to improve model sensitivity, to identify the magnitude of changes more precisely in regional complexity caused by aspects of PTSD in this sample. These measures serve to further our understanding of the underlying central neurobiological changes experiences by WTC responders with PTSD because of their traumatic exposures at 9/11.

## Materials and methods

Data derived from the Stony Brook–Mount Sinai WTC responder imaging study has been previously described [4]. Briefly, 99 participants were recruited from a single clinic-based WTC health monitoring program on Long Island, NY [69, 70], where an ongoing epidemiologic study of accelerated aging was undertaken [24]. PTSD diagnosis was determined from a structured diagnostic interview, described below. Inclusion criteria were ages 44 to 65, fluent in English, and a diagnostic assessment of WTC-PTSD. Subjects also satisfied eligibility criteria for MRI scanning including body mass index ≤40, no known claustrophobia, no known metal implants or shrapnel that was not deemed MRI-safe, and no prior history of traumatic brain injury. Upon enrollment, eligible responders were screened to ensure case status. The case and control groups were matched on age within 5 years, sex, race/ethnicity, type of responder (police vs nontraditional), and education. Overall, the 99 WTC responders who completed the study were mostly male (78.8%) and had an average age of 55.9 (SD 5.2).

### Imaging measures

#### Image acquisition

MRI acquisitions were performed on a 3 T Siemens Biograph mMR scanner at Icahn School of Medicine at Mount Sinai, NY. Three-dimensional T1-weighted magnetization-prepared rapid gradient echo (MPRAGE) images were acquired using the following parameters: TR = 1900ms, TE = 2.49 ms, TI = 900 ms; Flip Angle = 9°; acquisition matrix: 256 × 256; voxel resolution: 0.89 × 0.89 × 0.89 mm^3^. For incidental pathology screening, 2D T2-weighted anatomical scans used a turbo spin-echo pulse sequence (34 axial slices, TR = 6170 ms, TE = 96 ms; Refocusing flip angle = 150°; acquisition matrix = 320 × 320; pixel size = 0.36 × 0.36 mm^2^, slice thickness/gap = 3/0 mm), and were acquired and read by a board-certified radiologist to determine incidental findings.

#### Fractal Dimension image processing

Fractals are common in natural systems and identify structures that are nested within complex replications of their own surfaces [71]. The human brain is generally thought to be best described by 3D fractal geometry, instead of 2D Euclidean geometry; hence FD is a good utility to describe the complexity of the anatomical surface of the cortical ribbon [72].

Image processing was completed by analyzing 99 volumetric MRI images from WTC responders with (*n* = 47) and without (*n* = 52) PTSD and performing 3D fractal dimension (FD) analysis was conducted in Computational Analysis Toolbox 12 (CAT12, Structural Brain Imaging Group, University of Jena, Germany) in n Matlab R2019a version 9.6 (MathWorks Inc., Sherborn, MA, USA), which can extract fractal dimension values at the global (whole-brain), regional (based on regions of interest of an atlas), and local (vertex) level, based on a spherical harmonic reconstruction method previously described [73] and presently used. The CAT12 SBM toolbox provides detailed instructions for analysis [74] and prior work has found that CAT12 is reliable and highly efficient for segmentation purposes [75, 76]. To that end, structural images were first stratified in the left and right hemispheres before being resampled, merged, and smoothed at 25 mm following the recommended processing procedures [73]. Briefly, FD was estimated based on spherical reconstructions and was calculated as the slope of a logarithmic plot for surface area against the maximum l-value, which is the frequency bandwidth [73]. For regional analyses, ROI-based FD values were estimated for 68 unilateral ROIs using the Desikan–Killiany atlas included in the CAT12 toolbox using standard procedures [77]. Visual inspection of all images for motion artifacts and missing portions, and image rating was performed for quality control by two experts (S.C. and C.H.); all scans passed global visual inspection using the “Slice Display” and “Surface Data Homogeneity” tools in CAT12.

### Clinical measures

#### Posttraumatic stress disorder

PTSD diagnosis was determined using the Structured Clinical Interview for the DSM-IV [SCID-IV] [78], a semi-structured interview administered by trained clinical interviewers. Symptom subdomains were measured using subscales calculated using reported symptom severity in the SCID for the following symptom domains: reexperiencing symptoms, avoidance, hyperarousal, and negative life experiences.

### WTC exposure severity

WTC exposure severity was operationalized as time spent (in months) on site during the search and rescue efforts [25, 66].

### Statistical analyses

In the present study, confounding from key variables including age was completed using matching in the recruitment design phase. Pairwise t-tests with Welch’s correction and *χ*2 tabulations were used to examine differences in matching and diagnostic variables across diagnostic groups. Assessment of distribution for all continuous variables was performed with Shapiro–Wilk test and visualization of histogram densities. Statistical significance was considered when *p* < 0.05; Type I errors were controlled for by adjusting significance using the False Discovery Rate (FDR) set at 0.05 [79].

The analytic plan first focused on studying whole-brain surface-based morphometry (SBM) FD clusters. Whole-brain SBM analysis relied on threshold-free cluster enhancement algorithm (FCE; E = 0.5, H = 2.0) using Draper–Stoneman (10,000 permutations) to detect vertex clusters; FCE-calculated t-values were reported (*t*_FCE_) with *P* reporting a p-value that is adjusted for the false discovery rate. [80] Cluster-based analyses were reported and mapped onto central surface maps; cluster locations were reported in X/Y/Z indicate coordinates, expressed in millimeters, using the Montreal Neurological Institute’s coordinate system and then further remapped onto Desikan–Killiany ROIs for extended parcellation and visual comparison with the follow up ROI analysis (see below). Next, we examined absolute bilateral and unilateral FD, before finally examining FD in ROIs and employed two-tailed independent sample t-tests with Welch’s correction with effect sizes reported as Cohen’s *d*. Power analysis given two-tailed *α* = 0.05, at a minimum acceptable level of 80% power, with a sample size of *n* = 99, required an effect size of *d* = 0.57.

Next, we examined bivariate correlations between ROI FD, the four PTSD symptom cluster scores (reexperiencing, hyperarousal, avoidance, negative affect), and WTC exposure duration, using two-tailed Spearman’s rank correlation coefficients (*α* = 0.05). Power analysis given two-tailed *α* = 0.05, at a minimum acceptable level of 80% power, with a sample size of *n* = 99, required an effect size of Spearman’s Rho |*ρ*| = ± 0.27.

Finally, a series of multiple linear regressions were used, with each PTSD symptom cluster score (total combined, reexperiencing, hyperarousal, avoidance, negative affect) entered as predictors/independent variables, and each cortical ROI entered as outcome/dependent variables, while adjusting for cortical thickness in each respective ROI, major depressive disorder, anxiety disorder, diabetes, sleep apnea, obstructive airway disease, gastroesophageal disorder, and cardiovascular disease.

Analyses were completed in: JASP (Version 0.15.0), JASP Team (2020), University of Amsterdam, Netherlands; GraphPad Prism (Version 9.1.0), GraphPad Software, San Diego, California USA; and G*Power 3.1, IDRE Stats, UCLA.

### Ethics

The Institutional Review Board at both Stony Brook University and the Icahn School of Medicine at Mount Sinai approved study procedures; participants provided informed written consent.

## Results

By design, sample subgroups were matched in terms of age at scan, sex, race/ethnicity, educational attainment, and analyses reported below indicate that the matching was successful. Therefore, analyses did not require additional adjustments for these matching variables.

### FD in WTC-PTSD

We first compared responders with and without DSM-IV WTC-PTSD on background and testing characteristics. Table 1 shows that responders with a diagnosis of WTC-PTSD were not demographically different from those without PTSD, consistent with the matching design of the study. Nor were there differences in duration of efforts at the WTC recovery site. As expected, responders with a clinical diagnosis of PTSD reported significantly greater PTSD symptom severity on all subscales. Furthermore, significantly more responders with WTC-PTSD had higher rates of comorbidities (see Table 1).

**Table 1:** Sample (*n* = 99) characteristics for WTC responders with and without PTSD diagnosis.

Characteristic	PTSD- (*n* = 52)	PTSD + (*n* = 47)	*p*
Age	56.88 (5.31)	55.81 (5.05)	0.3073
BMI	29.55 (4.03)	28.86 (5.04)	0.3943
*Sex*			0.6331
Male	76.92%	80.85%	
Female	23.08%	19.15%	
*Race*			0.8724
Black	11.54%	8.51%	
White	75.00%	78.72%	
Other	13.46%	12.77%	
*Hispanic*			0.2953
Yes	15.38%	8.51%	
No	84.62%	91.49%	
Education (school years)	15.71 (1.96)	15.28 (2.47)	0.3391
*WTC Exposures*			
WTC efforts (months)	3.75 (3.19)	3.69 (2.49)	0.9373
Engulfed by 9/11 dust cloud	11.54%	12.77%	0.8518
Early arrival 9/11 - 9/12	78.85%	72.34%	0.4507
Exposure to human remains	63.46%	74.47%	0.2383
Experienced loss of life	53.85%	76.60%	0.0181*
Experienced suffering of others	53.85%	72.34%	0.0575
*DSM-IV SCID Trauma Screen*			
Total PTSD symptoms	49.62 (8.08)	95.81 (13.03)	3.9639e -33*
Reexperiencing	12.17 (3.12)	23.36 (4.29)	7.2437e -25*
Avoidance	16.27 (3.23)	32.70 (6.13)	2.1250e -25*
Hyperarousal	12.13 (2.92)	23.98 (3.33)	2.6739e -32*
Negative affect	9.04 (1.66)	15.77 (4.19)	8.2934e -15*
*Comorbidities*			
Major Depressive Disorder	0.00%	38.30%	8.0727e -7*
Anxiety Disorder	9.62%	31.92%	0.0058*
Substance Abuse Disorder	1.92%	2.13%	0.9424
Diabetes	3.00%	9.00%	0.0417*
Hypertension	17.00%	18.00%	0.5602
Sleep Apnea	21.15%	55.32%	0.0005*
Upper Respiratory Disease	61.54%	68.09%	0.4963
Obstructive Airway Disease	30.77%	53.19%	0.0237*
GERD	32.69%	63.83%	0.0019*
Coronary Artery Disease	0.00%	6.38%	0.0643
Heart Attack	1.92%	4.26%	0.4990
Abnormal Heartbeat	0.00%	6.38%	0.0643
Heart Murmur	1.92%	6.38%	0.2604
Congestive Heart Disease	1.92%	0.00%	0.3393
Other Heart Disease	9.62%	21.28%	0.1061
Cardiovascular Disease	15.38%	38.30%	0.0097*
Cancer	19.23%	25.53%	0.4514

Note: Means (standard deviations) or percentages (%) reported, *P*-values examine the extent to which noted characteristics differ and were derived using *χ*2 tests for categorical variables, and independent samples Welch’s *t*-tests for continuous variables, *denotes significance of *p* < 0.05, BMI body mass index, PTSD posttraumatic stress disorder, DSM-IV SCID structured clinical interview for DSM disorders, GERD gastroesophageal reflux disease, WTC world trade center.

**Fig. 1:**
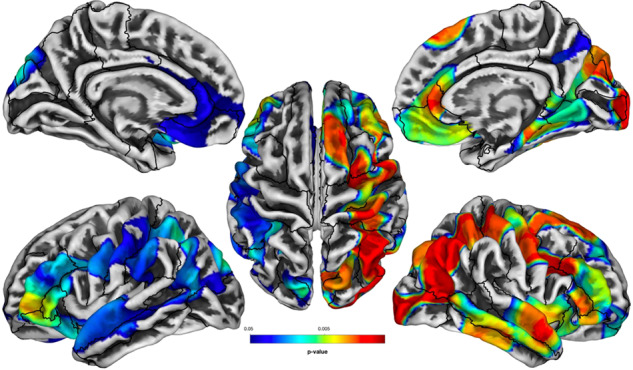
Group-wise nonparametric analyses of FD using surface-based morphometry comparing PTSD negative to PTSD positive responders. Regions lacking significant differences in FD between groups are shown in gray while statistically significant results are shown using a rainbow colormap ranging from blue to red. Figure generated using Computational Anatomy Toolbox (CAT12) with superimposed Desikan–Killiany (DK) atlas regions shown in black borders.

We then performed SBM analyses of cortical FD clusters in WTC responders with/without PTSD and identified statistically significant clusters with five cluster-peaks that survived adjustment for multiple comparisons [see Fig. 1]. Cluster-peaks were evident predominantly in the right hemisphere with focal alterations in the parietal lobe reaching into the superior temporal gyrus. The most significant reductions in FD were focused across several large clusters including a focal peak in the right superior parietal (MNI coordinates: 32/−52/54 mm, tTFCE = 17,521.41, *p* = 0.002), the left frontal pole (−39/43/−2; tTFCE = 7798.88, *p* = 0.001), the left lateral occipital (−13/−87/29; tTFCE = 4749.16, *p* = 0.002), the right precentral (6/−29/74; tTFCE = 2239.24; *p* = 0.050), the left anterior cingulate (−3/−2/36; tTFCE = 1,639.78; *p* = 0.050), and the left caudal anterior cingulate (−4/5/30; tTFCE = 1592.69; *p* = 0.050).

**Fig. 2:**
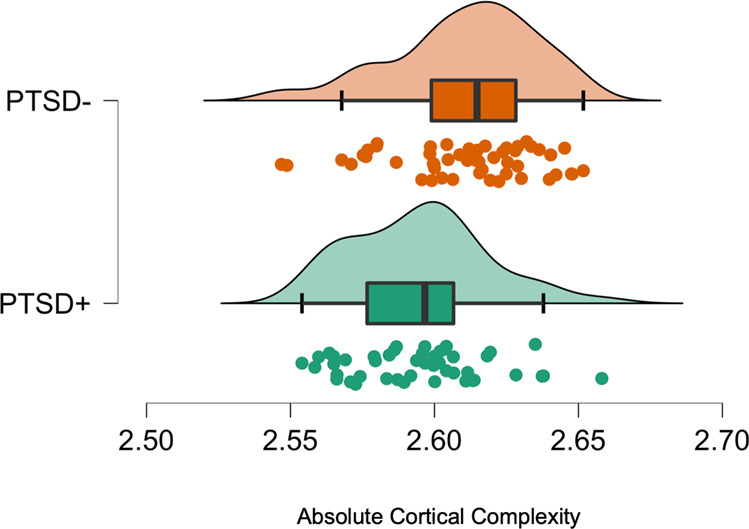
Raincloud plots showing data distribution (cloud), jittered raw data and central tendency boxplots comparing absolute cortical complexity between WTC responders with PTSD versus those without. Analyses identified significantly lower absolute whole brain cortical complexity (Welch’s *t* = −3.3144, *p* = 0.0013, Cohen’s d = −0.67) in the PTSD + group (mean = 2.5946, SD = 0.0234), when compared to the PTSD- group (mean = 2.6107, SD = 0.0247). PTSD posttraumatic stress disorder, X-axis denotes whole brain FD.

Whole brain analysis of FD using pairwise comparisons identified significantly lower absolute FD in the PTSD + group (*t* = −3.3144, *p* = 0.0013, mean difference −0.0160, *SE* = 0.0048, Cohen’s d = −0.67) [see Fig. 2].

**Fig. 3:**
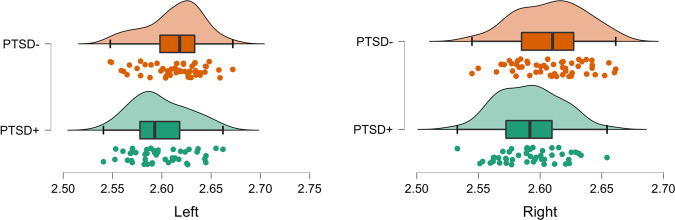
Raincloud plots showing pairwise comparisons between bilateral cortical complexity between WTC responders with PTSD versus those without. Analyses identified lower absolute cortical complexity in the PTSD + group in the left hemisphere (*t* = −2.5535, *p* = 0.0122, Cohen’s d = −0.5139; PTSD+: mean = 2.5979, SD = 0.0290; PTSD−: mean = 2.6128, SD = 0.0289), and the right hemisphere (*t* *=* *−*3.1798, *p* = 0.0020, Cohen’s d = −0.6358; PTSD + : mean = 2.5913, SD = 0.0257; PTSD−: mean = 2.6085, SD = 0.0282), when compared to the PTSD- group. PTSD posttraumatic stress disorder, *X*-axis denotes hemispheric FD.

Examination of unilateral hemispheric differences in FD using pairwise comparisons identified significantly lower absolute unilateral FD in the left hemisphere (*t* = −2.5535, *p* = 0.0122, mean difference −0.0149, *SE* = 0.0058, Cohen’s d = −0.5139), with a larger reduction in the right hemisphere (*t* = −3.1798, *p* = 0.0020, mean difference −0.0172, *SE* = 0.0054, Cohen’s d = −0.6358) in the PTSD + group when compared to the PTSD- group (see Fig. 3).

Examination of regional differences using pairwise comparisons of FD in each of the 68 unilateral ROIs identified nominally significantly lower cortical complexity in the PTSD + when compared to the PTSD− group. However, given adjustment for 68 multiple comparisons, none survived FDR, which we suspect as a Type-II error. Therefore, we report the nominally significant reductions spanning across the frontal, temporal, and parietal lobe (see Table 2).

**Table 2:** Pairwise comparisons of regional fractal dimension (FD) cortical complexity using independent samples *t*-test with Welch’s correction between WTC responders with/without PTSD.

		PTSD− (*n* = 52)		PTSD+ (*n* = 47)				
Lobe	Regional complexity	Mean	SD	Mean	SD	*t*	*p*	*d*
Frontal	*L Pars Triangularis*	2.759	0.158	2.695	0.161	−1.988	0.05	−0.4
	*R Pars Triangularis*	2.736	0.14	2.677	0.146	−2.041	0.044	−0.41
	*R Precentral*	2.766	0.077	2.737	0.063	−2.113	0.037	−0.42
Temporal	*R Middle Temporal*	2.644	0.085	2.596	0.11	−2.422	0.018	−0.49
	*R Superior Temporal*	2.964	0.115	2.915	0.108	−2.196	0.031	−0.44
	*R Inferior Parietal*	2.783	0.095	2.741	0.088	−2.287	0.024	−0.46
Parietal	*R Superior Parietal*	2.711	0.091	2.666	0.093	−2.478	0.015	−0.5
	*L Supramarginal*	2.679	0.079	2.645	0.09	−2.029	0.045	−0.41

Note: nonsignificant results are omitted; SD standard deviation, *t* statistic, *p* < 0.05, *d* = Cohen’s d effect size.

Associations between dimensional measures of PTSD symptom clusters and WTC exposure duration (months) with FD in each ROI identified multiple significant correlations between reexperiencing symptom cluster and the right pars triangularis, the right rostral middle frontal and the right paracentral. We also found significant associations between the avoidance cluster and the right pars triangularis, the right rostral middle frontal and the right postcentral, and between the negative affect cluster and the left and right superior parietal (see Supplementary Table 1).

A series of adjusted multiple regression models were conducted to estimate the effects of total PTSD symptom severity along with the individual four PTSD symptom severity clusters, with each entered individually as the independent variable/predictor on each ROI FD serving as the dependent variable/outcome. Analyses identified significantly negative estimates across four regions in the frontal lobe, three regions in the limbic lobe, two regions in the temporal lobe and seven regions in the parietal lobe (see Table 3).

**Table 3:** Series of multiple linear regressions, adjusted for cortical thickness in respective region, major depressive disorder, anxiety disorder, diabetes, sleep apnea, obstructive airway disease, gastroesophageal disorder, and cardiovascular disease. Predictors entered in the model were either total PTSD score or a single PTSD symptom score (Reexperiencing, Avoidance, Hyperarousal or Negative affect) with a single FD cortical region (from 68 unilateral regions of interest parcellated from the Desikan–Killiany atlas) entered as outcome.

	Dependent variable/	Independent variable/predictor																			
	Outcome	Total PTSD symptoms				Re-experiencing				Avoidance				Hyperarousal				Negative affect			
Lobe	Cortical Region FD	*B*	*SE B*	*β*	*P*	*B*	*SE B*	*β*	*P*	*B*	*SE B*	*β*	*P*	*B*	*SE B*	*β*	*P*	*B*	*SE B*	*β*	*P*
Frontal	*L Frontal Pole*	−0.002	0.136	−0.211	0.049					−0.005	0.002	-0.213	0.046								
	*R Frontal Pole*																	−0.010	0.039	−0.264	0.015
	*R Pars Triangularis*	−**0.001**	**0.001**	−**0.239**	**0.022**	−**0.006**	**0.002**	−**0.299**	**0.004**	−0.003	0.002	−0.217	0.038								
Limbic	*R Parahippocampal*					−0.005	0.003	−0.190	0.043												
	*R Isthmus Cingulate*																	−0.005	0.002	−0.239	0.025
Temporal	*R Middle Temporal*	**−0.001**	**0.000**	−**0.256**	**0.017**	−**0.003**	**0.002**	−**0.227**	**0.037**	−**0.003**	**0.001**	−**0.242**	**0.024**					**−0.006**	**0.002**	−**0.280**	**0.005**
	*R Superior Temporal*	−0.001	0.001	−0.210	0.044													−0.007	0.003	−0.268	0.011
Parietal	*L Inferior Parietal*	−0.001	0.000	−0.215	0.034					−0.003	0.001	−0.226	0.026	−0.004	0.002	−0.236	0.018				
	*R Inferior Parietal*																	−0.005	0.002	−0.229	0.032
	*R Paracentral*					0.004	0.002	0.254	0.018					0.003	0.002	0.222	0.036				
	*R Postcentral*	−0.001	0.000	−0.227	0.027					−0.003	0.001	−0.250	0.014					−0.005	0.003	−0.219	0.036
	*L Superior Parietal*																	−0.004	0.002	−0.231	0.022
	*R Superior Parietal*	**−0.001**	**0.000**	−**0.254**	**0.016**				**0.124**	**−0.003**	**0.001**	−**0.262**	**0.013**	**−0.003**	**0.001**	−**0.226**	**0.031**	**−0.007**	**0.002**	**−0.331**	**0.001**

Note: Nonsignificant findings are omitted; values in BOLD = survived multiple comparisons adjustment at FDR = 0.05; nominally significant values at *p* < 0.05 are shown without bold; *B* = unstandardized estimate; *SE B* = standard error of *B*; *β* = standardized estimate.

## Discussion

In this study, we tested the hypothesis that reductions in cerebral complexity would be found in individuals with chronic PTSD in a sample of 99 WTC responders assessed almost two decades after 9/11. We confirmed that reductions in cortical complexity, previously found in specific psychiatric disorders, were also noted in WTC responders with PTSD. Using FD to measure both global, unilateral, and regional cortical complexity, we identified significant associations between reduced FD and WTC-PTSD. SBM analysis revealed clusters that were significantly reduced in PTSD + responders across the frontal, parietal, temporal, and occipital lobes.

Examination of hemispheric cortical complexity also identified reductions like those identified with SBM analysis. The right hemisphere was found to have a larger reduction in cortical complexity in WTC responders with PTSD when compared to matched WTC responders without PTSD. Prior studies have demonstrated larger activations in the right hemisphere in traumatized populations [81–84]. The right hemisphere develops earlier than the left, and has been implicated in nonverbal emotional communication and the dynamic and holistic integration across sensory modalities [85]. The right hemisphere also greatly integrates with the amygdala, which processes emotional significance of sensory inputs and then outputs appropriate hormonal and autonomic responses, and has been implicated with personality, emotional, and behavioral disturbances [86, 87]. In contrast, the left hemisphere, which is largely involved with verbal communication and problem-solving abilities, seems to be less associated with PTSD [88]. Therefore, our results identifying greater reductions in cortical complexity of the right hemisphere in responders with WTC-PTSD are largely consistent with the literature, in so far that the right hemisphere appears to be more generally affected by PTSD when compared to the left hemisphere, which may have led to the observed reduction in right hemisphere complexity.

Similar to the present findings, SBM reductions of FD in the superior parietal, the frontal pole, the precentral gyrus, the lateral occipital and the cingulate gyri have been reported in psychiatrically affected populations with anorexia nervosa, with a reduction of FD in the parietal lobe, which is involved in self-image and body-image perception, suggesting that this region may be particularly vulnerable to changes in cortical complexity in psychiatric conditions [46]. Eating disorders share similarities with PTSD regarding the rates of dissociative behaviors, where an increase of traumatic events in an individual’s life can lead to higher rates of developing eating disorders [89]. Moreover, patients with negative schizophrenia have also been identified to display reductions in FD in the superior parietal, the frontal pole, precentral gyrus, and cingulate gyri [47]. Negative schizophrenia and PTSD share certain psychiatric similarities in so far that they can both exhibit negative symptoms of social withdrawal, loss of volition, affective flattening, and poverty of speech, presumed to be associated with structural brain abnormalities [90]. Given the evidence and the overlap in regions displaying FD reductions between these two conditions, these brain regions may be susceptible to changes in cortical complexity in psychiatrically complex populations.

Segmentation identified nominally significant, but reduced FD in regions spanning across the frontal, temporal, and parietal lobes in WTC responders with chronic PTSD. Multiple regression models entering total PTSD symptom severity and individual symptom severity as the predictor, with FD from individual ROIs as the outcome, while adjusting for CTX in the respective region, revealed significant negative effects of total, and individual PTSD symptoms in ROIs spanning across the frontal, temporal, and parietal lobes. The regions with FD reductions identified in this study encompass cortical areas involved in: language control [91]; executive function; emotion regulation and working memory [92, 93]; perceived stress [94, 95]; PTSD trauma [96–98]; chronic schizophrenia [99, 100]; and cognitive decline [101–105]. The significant associations of PTSD symptoms with the parietal lobe, such as negative experiences associated with reduced FD in the postcentral gyrus (which is the major component of the primary somatosensory cortex), have been implicated with exposure to trauma [106], decreased inhibition in PTSD combat veterans [107], as well as a positive association of resting state activity within this region with the number of traumatic experiences [108]. Furthermore, clusters of associations were observed in the superior parietal lobule, which has been implicated with reduced cortical thickness in miners with PTSD [96, 109], the dissociative subtype of PTSD [110], greater fMRI activation among PTSD patients [111], and reduced cortical complexity in patients with negative and paranoid schizophrenia [47]. Taken together, these regional findings suggest that WTC-PTSD from exposure to traumatic events during the search and rescue efforts at 9/11 may have led to reductions in cortical complexity. It is also possible that prior reduction in cortical complexity may have predisposed responders to PTSD.

SBM analysis identified clusters that were significantly reduced in responders with PTSD, with peaks included from areas such as the left frontal pole, the left lateral occipital, the left anterior cingulate, and the left caudal anterior cingulate, that were not replicated in the ROI analysis, while the focal peak in the right precentral was. The reason for this discrepancy may lie in the fact that ROI based analyses are univariate approaches that are selected based on atlases using prior knowledge that do not always fully account for anatomical brain differences in individuals [112, 113]. In contrast, SBM analyses represent a multivariate approach that identifies peaks or troughs in a specific measure and can identify clusters around those areas that are correspondingly affected. Peaks/troughs are identified at the node in SBM, while the cluster may range in any direction from the peak. This has the advantage of being anatomically unrestricted and better able to capture even small spatial differences between groups of individuals as it provides maps which display common covariation where changes are evident [113, 114], though changes should be smaller in some portions of the cluster than in others potentially leading to small differences between conclusions made using SBM as compared to ROI-based analyses.

Cortical complexity is a developmental process, whereby the increase in folding of the cortical ribbon begins at the neonatal stage and increases with development and life experience. It is also associated with cortical thickness and neural density and has been previously demonstrated to be negatively associated with neurological and psychiatric disorders, and the aging brain. However, very little is known about the etiology of reduced cortical complexity in WTC responders with chronic PTSD, let alone the association of cortical complexity with PTSD in other trauma affected populations. As has been previously noted, it has been demonstrated that PTSD is a risk factor for CI and that PTSD is associated with neurobiological changes, such as a reduction in cortical thickness. However, our prior neuroimaging study of cortical thickness in WTC responders with PTSD failed to show significant reductions in cortical thickness associated with PTSD. Alternatively, recent studies have shown a reduction of gyrification in patients with PTSD, and several recent studies in psychiatry have demonstrated distinct patterns of changes in cortical complexity, suggesting that PTSD may be associated with morphological changes at the cerebral surface. Therefore, to better interrogate the underlying neurobiological changes in WTC responders with long-term PTSD, we employed the novel measure of FD for cortical complexity and identified several reductions associated with PTSD. We are, however, in part limited by the prior literature, which is nascent due to a reliance on this relatively novel measurement.

According to the tension-based hypothesis, cortical complexity during development is associated with elongation or retraction of axonal tension, which in turn leads to the formation of gyri and sulci, thereby influencing complexity of the cortical surface [115–118]. Thus, cortical complexity can be influenced by underlying white matter connectivity, whereby disruptions in the connectivity of underlying axons could reduce surface complexity. Indeed, studies have documented disruptions in white matter connectivity and integrity in trauma and PTSD populations [119–124], which has been proposed to have an underlying etiology due to increased neuroinflammation due to psychosocial stress [125–132], with accelerated brain senescence [133–135], and that normal brain aging is associated with reduced cortical complexity [39, 58–61]. Coupled with our own studies that have identified increased neuroinflammation in responders with chronic WTC-PTSD [68, 136–138], we therefore propose that one possible avenue for the reduced cortical complexity observed in WTC responders with chronic PTSD in this study may be due to increased neuroinflammation from elevated psychosocial stress, which may have led to white matter atrophy, in turn leading to reduced cortical complexity through retraction of axonal tension. Future studies with WTC responders and other trauma affected populations should directly interrogate FD analysis, and if possible, integrate diffusion tensor imaging analyses of white matter integrity from MRI, along with concurrent Positron Emission Tomography (PET) biomarkers of neuroinflammation, such as the Translocator protein 18-kDa (TSPO) ligand FEPPA, or others. Doing so, would better position future research studies to interrogate this theory and further investigate the neurobiological mechanisms involved in the reduction of cortical complexity, whether in WTC responders with PTSD, or with other trauma exposed populations with PTSD. Such studies may further elucidate why PTSD is a risk factor CI.

The present study is the first study to investigate this novel marker of cortical complexity in a sample of WTC responders and the first study to investigate this measure specifically in WTC-PTSD. Our findings not only identify that cortical complexity was negatively associated with WTC-PTSD, but also provide evidence that reduced cortical complexity may be negatively associated with PTSD as a psychiatric condition. Future neuroimaging studies interrogating the neural correlates of PTSD with a non-WTC exposed control group without PTSD will be better able to generalize their results by quantifying cortical complexity in addition to gray matter volumetric, cortical thickness, and functional MRI approaches. Such approaches may prove fruitful to future research studies of trauma and PTSD affected populations to help understand the presence, prognosis, and features of related psychiatric symptoms that aid clinical monitoring of such individuals. Detection of novel characteristic neuroanatomical differences in psychiatric patients will lead to more effective diagnosis, treatment, and ultimately interventions. Since we did not find evidence of changes in cortical thickness associated with PTSD in our WTC responder population, but instead identified reductions in the complexity of the cortical ribbon associated with PTSD, future studies should test whether cortical complexity is a signature in other PTSD populations, and if so, whether it serves as a biomarker for PTSD.

### Limitations

While novel in many ways, this study has a number of important limitations including a small sample size, unique nature of WTC exposures, and lack of non-WTC external control group that together prevent us from generalizing our findings to the general population. Though we did make efforts to increase recruitment of minorities and women to the point of doubling the numbers of both in this sample compared to the responder population enrolled in our program, the sample would benefit from improved diversity. Furthermore, although we found significant differences between those with and without PTSD, we could not shed light on time of onset and possible changes in cortical complexity in responders with WTC-PTSD. Specifically, the finding of reduced FD in responders with WTC-PTSD could have resulted from a one-time massive systemic shock or cumulative effects of chronic stressors leading to PTSD. In addition, we do not have accurate assessments of life trauma and/or PTSD in WTC responders prior to 9/11, and we lack a comparison group of responders with sub-syndromal, mild, heterogeneous, or remitted PTSD. While current medication yes/no is collected at the WTC-HP for responders with comorbidities, they were unfortunately not in the analysis files, and we hope to analyze medications (medical and psychiatric) in future papers. Finally, PTSD symptom clusters of reexperiencing, avoidance, negative affect, and hyperarousal are strongly inter-correlated. Hence disentangling the unique contribution of each symptom cluster to changes in cortical complexity is challenging and will require larger samples, and samples with greater symptom heterogeneity, in the future. Nevertheless, our results still demonstrate a clear association between WTC-PTSD and reduced cortical complexity.

## Conclusions

This is the first study of its kind to examine cortical complexity in a sample of WTC responders. The WTC disaster exposed tens of thousands of individuals, including professional and civilian responders, to the toxic detritus of the towers after they collapsed and the ensuing emotional disturbances arising from the terrorist attacks. The most prominent psychiatric conditions among exposed responders have been chronic PTSD with a prevalence of approximately 23%. Our findings herein report evidence that WTC-PTSD is negatively associated with a novel marker of cortical complexity across different cortical areas, extending previous studies of other psychiatric disorders. Thus, our study is the first to study cortical complexity in a population with and without chronic PTSD and the first to demonstrate that PTSD is associated with reductions in cortical complexity. More research is needed to determine if the current findings are replicated in other, non-WTC trauma-exposed populations and if neuroimaging measures of cortical complexity, such as fractal dimension, serve as a biomarker for PTSD in trauma- affected individuals.

## Supplementary information

Supplemental Table 1

## Data Availability

Data can be made available by request to the corresponding author.
