# Supplementary material for: Cortical complexity in world trade center responders with chronic posttraumatic stress disorder
Source: Transl Psychiatry. 2021 Nov 23;11:597. doi: 10.1038/s41398-021-01719-7 (PMC8611009; doi:10.1038/s41398-021-01719-7)
Supplement: Supplementary file 1 — Supplemental Table 1 [file 41398_2021_1719_MOESM1_ESM.docx]

|  | **Region of interest** | **Re-Experiencing** | **Avoidance** | **Hyperarousa** | **Negative Affect** | **WTC Exposure Duration** |
| --- | --- | --- | --- | --- | --- | --- |
|  | *L Caudal Middle Frontal* |  |  |  |  |  |
|  | *R Caudal Middle Frontal* |  |  |  |  |  |
|  | *L Frontal Pole* |  |  |  |  |  |
|  | *R Frontal Pole* |  |  |  |  |  |
|  | *L Lateral Orbitofrontal* |  |  |  |  |  |
|  | *R Lateral Orbitofrontal* |  |  |  |  |  |
|  | *L Medial Orbitofrontal* |  |  |  |  |  |
|  | *R Medial Orbitofrontal* |  |  |  |  |  |
| **Frontal** | *L Pars Opercularis* |  |  |  |  |  |
| **Lobe** | *R Pars Opercularis* |  |  |  |  |  |
|  | *L Pars Orbitalis* |  |  |  |  |  |
|  | *R Pars Orbitalis* |  |  |  |  |  |
|  | *L Pars Triangularis* |  |  |  |  |  |
|  | *R Pars Triangularis* | -0.33 | -0.28 |  |  |  |
|  | *L Rostral Middle Frontal* |  |  |  |  |  |
|  | *R Rostral Middle Frontal* | -0.33 | -0.28 |  |  |  |
|  | *L Superior Frontal* |  |  |  |  |  |
|  | *R Superior Frontal* |  |  |  |  |  |
|  | *L Precentral* |  |  |  |  |  |
|  | *R Precentral* |  |  |  |  |  |
|  | *L Caudal Anterior Cingulate* |  |  |  |  |  |
|  | *R Caudal Anterior Cingulate* |  |  |  |  |  |
|  | *L Rostral Anterior Cingulate* |  |  |  |  |  |
|  | *R Rostral Anterior Cingulate* |  |  |  |  |  |
|  | *L Isthmus Cingulate* |  |  |  |  |  |
| **Limbic** | *R Isthmus Cingulate* |  |  |  |  |  |
| **Lobe** | *L Insula* |  |  |  |  |  |
|  | *R Insula* |  |  |  |  |  |
|  | *L Parahippocampal* |  |  |  |  |  |
|  | *R Parahippocampal* |  |  |  |  |  |
|  | *L Posterior Cingulate* |  |  |  |  |  |
|  | *R Posterior Cingulate* |  |  |  |  |  |
|  | *L Banks Superior Temporal Sulcus* |  |  |  |  |  |
|  | *R Banks Superior Temporal Sulcus* |  |  |  |  |  |
|  | *L Entorhinal* |  |  |  |  |  |
|  | *R Entorhinal* |  |  |  |  |  |
|  | *L Inferior Temporal* |  |  |  |  |  |
|  | *R Inferior Temporal* |  |  |  |  |  |
| **Temporal** | *L Middle Temporal* |  |  |  |  |  |
| **Lobe** | *R Middle Temporal* |  |  |  |  |  |
|  | *L Superior Temporal* |  |  |  |  |  |
|  | *R Superior Temporal* |  |  |  |  |  |
|  | *L Temporal Pole* |  |  |  |  |  |
|  | *R Temporal Pole* |  |  |  |  |  |
|  | *L Transverse Temporal* |  |  |  |  |  |
|  | *R Transverse Temporal* |  |  |  |  |  |
|  | *L Inferior Parietal* |  |  |  |  |  |
|  | *R Inferior Parietal* |  |  |  |  |  |
|  | *L Paracentral* |  |  |  |  |  |
|  | *R Paracentral* | 0.27 |  |  |  |  |
| **Parietal** | *L Postcentral* |  |  |  |  |  |
| **Lobe** | *R Postcentral* |  | -0.30 |  |  |  |
|  | *L Precuneus* |  |  |  |  |  |
|  | *R Precuneus* |  |  |  |  |  |
|  | *L Superior Parietal* |  |  |  | -0.27 |  |
|  | *R Superior Parietal* |  |  |  | -0.30 |  |
|  | *L Supramarginal* |  |  |  |  |  |
|  | *R Supramarginal* |  |  |  |  |  |
|  | *L Pericalcarine* |  |  |  |  |  |
|  | *R Pericalcarine* |  |  |  |  |  |
|  | *L Fusiform* |  |  |  |  |  |
|  | *R Fusiform* |  |  |  |  |  |
| **Occipital** | *L Cuneus* |  |  |  |  |  |
| **Lobe** | *R Cuneus* |  |  |  |  |  |
|  | *L Lateral Occipital* |  |  |  |  |  |
|  | *R Lateral Occipital* |  |  |  |  |  |
|  | *L Lingual* |  |  |  |  |  |
|  | *R Lingual* |  |  |  |  |  |

**Supplementary table 1.** Heat map showing magnitudes of significant Spearman’s correlation coefficients (*r_s_*) examining dimensional measures of posttraumatic stress disorder symptoms and measures of fractal dimensionality (FD) in 68 unilateral cortical regions of interest segmented from a brain atlas. Nominal significance was set at: *P* < .05 at 80% power, with *ρ* = ±0.27, n = 99. Note: Non-significant values or underpowered are omitted; red indicates negative association; blue indicates positive association. Abbreviations: WTC = World Trade Center; L = left hemisphere; R = right hemisphere.
